# Myocytes Oxygenation and High Energy Phosphate Levels during Hypoxia

**DOI:** 10.1371/journal.pone.0101317

**Published:** 2014-09-30

**Authors:** Mohammad Nurulqadr Jameel, Qingsong Hu, Jianyi Zhang

**Affiliations:** Departments of Medicine, Radiology, the Center for Magnetic Resonance Research, and University of Minnesota Health Sciences Center, University of Minnesota, Minneapolis, Minnesota, United States of America; Northwestern University, United States of America

## Abstract

Decrease of ambient oxygen level has been used in myocytes culture experiments in examining the responsiveness to stress secondary to hypoxia. However, none of these studies measure the myocytes oxygenation levels resulting in ambiguity as to whether there is insufficient oxygen delivery. This study examined the hypothesis that at a basal myocardial work state, adequate myocyte oxygenation would be maintained until extremely low arterial pO_2_ levels were reached. Myocyte pO_2_ values in normal dogs were calculated from the myocardial deoxymyoglobin (Mb- δ) levels using ^1^H-spectroscopy (MRS) and were normalized to Mb-δ obtained after complete LAD occlusion. During Protocol 1 (n = 6), Mb-δ was measured during sequential reductions of the oxygen fraction of inspired gas (FIO_2_) from 40, 21, 15, 10, and 5%, while in protocol 2 (n = 10) Mb-δ was measured at FIO_2_ of 3%. Protocol 3 (n = 9) evaluated time course of Mb-δ during prolonged exposure to FIO_2_ of 5%. Myocardial blood flow (MBF) was measured with microspheres and high energy phosphate (HEP) levels were determined with ^31^P-MRS. MVO_2_ progressively increased in response to the progressive reduction of FIO_2_ that is accompanied by increased LV pressure, heart rate, and MBF. Mb-δ was undetectable during FIO_2_ values of 21, 15, 10, and 5%. However, FIO_2_ of 3% or prolonged exposure to FIO_2_ of 5% caused progressive increases of Mb-δ which were associated with decreases of PCr, ATP and the PCr/ATP ratio, as well as increases of inorganic phosphate. The intracellular PO_2_ values for 20% reductions of PCr and ATP were approximately 7.4 and 1.9 mmHg, respectively. These data demonstrate that in the in vivo system over a wide range of FIO_2_ and arterial pO_2_ levels, the myocyte pO_2_ values remain well above the K_m_ value with respect to cytochrome oxidase, and oxygen availability does not limit mitochondrial oxidative phosphorylation at 5% FIO_2_.

## Introduction

In cell culture studies, the different levels of reduction in oxygen in the ambient air are often used as an intervention to examine the hypoxia induced changes of the respective cell types being examined [2], [28]. However, none of these studies measure the intracellular oxygenation levels, resulting in ambiguity as to whether there is insufficient oxygen delivery. In the in vivo studies, graded hypoxia is associated with relatively unchanged systemic hemodynamics and high energy phosphates when fraction of inflow oxygen (FIO_2_) is maintained above 8% [23]. However, more severe hypoxia (FIO_2_<6%) is accompanied by progressively more severe decreases in myocardial high-energy phosphates (HEP) and increases in inorganic phosphate [19]. This occurs despite the fact that myocardial oxygen consumption is maintained (by an increase in blood flow) throughout the hypoxic period [19]. Portman *et al* determined a critical arterial pO_2_ of 17–20 mmHg below which a decrease in high energy phosphate was noted [19]. They postulated that at this level, myocardial respiration probably becomes dependent on cellular oxygen supply because of a decreased capillary to mitochondrial gradient, although the intracellular oxygen tension was not measured.

^1^H nuclear magnetic resonance spectroscopy can be used to detect myoglobin desaturation. The unpaired electron spin in the heme-Fe(II) complex of deoxymyoglobin (Mb-δ) extends over the proximal histidyl N_δ_ proton to cause a chemical shift that produces a characteristic resonance on ^1^H NMR spectroscopy [6]. Using isolated perfused rat hearts, Kreutzer and Jue were the first to demonstrate that the degree of myoglobin deoxygenation can be quantitated with NMR spectroscopy and used to determine intracellular oxygen tension [12]. They observed that the phosphocreatine (PCr) level began to decrease at an intracellular pO_2_ of approximately 2 mmHg and fell to 80% of the basal value at an intracellular pO_2_ of 1.8 mmHg [12]. Further reductions of perfusate oxygenation resulted in sharp further decreases of PCr [12]. Since these data were obtained in isolated hearts perfused with non-hemoglobin containing buffer at 25°C, our laboratory has adapted this technique for in vivo use and has shown that decreases of myocardial blood flow as a result of graded coronary artery stenoses results in an increasing Mb-δ signal that is proportional to the decrease in blood flow [3], [27]. With the assumption that myoglobin was 90% saturated with O_2_ during basal conditions and 5% saturated during total coronary occlusion, the intracellular pO_2_ values for 20% reductions of PCr and ATP were calculated to be approximately 4.4 and 0.9 mmHg respectively [27]. However, the critical intracellular pO_2_ where significant decreases in high energy phosphates occurred could have been influenced by a decrease in myocardial oxygen consumption caused by a decrease in coronary flow and perfusion pressure [7]. This flow-related decrease in energy demand would allow the heart to accommodate to the decreased oxygen availability during hypoperfusion. Thus, stenosis induced hypoperfusion leads to decreased myocardial oxygen consumption through signals that decrease contractile activity or even by signals that directly inhibit mitochondrial respiration. The decreased oxygen consumption allows the myocardium to maintain HEP levels at lower intracellular PO_2_ levels. Thus, the true critical PO_2_ level that leads to a reduction of HEP can be better estimated with graded hypoxia through decrease in FIO_2_ such as in the present experimental setting.

Consequently, the present study was carried out to examine the effect of decreasing myocyte oxygenation by graded hypoxia on myocardial high energy phosphate content in the intact heart *in vivo*. Graded reductions of FIO_2_ were produced to document the threshold level of myocyte oxygenation at which perceptible reductions of the PCr/ATP ratio (corresponding to an increase of myocardial free ADP) occurred, and to examine the effect of progressive reductions of myocyte oxygenation on myocardial high energy phosphate content.

## Methods

Studies were performed in 25 adult mongrel dogs of either sex weighing 20–27 kg. All experimental procedures were approved by the University of Minnesota Animal Resources Committee. The investigation conformed to the “Guide for the Care and Use of Laboratory Animals” published by the US National Institutes of Health [NIH publication #85–23, revised 1985].

### Experimental preparation

The dogs were anesthetized with sodium pentobarbital (30–35 mg/kg bolus followed by 4 mg/kg/hr, i.v.), intubated and ventilated with a respirator with supplemental oxygen to maintain arterial blood gases within the physiologic range. A heparin-filled polyvinyl chloride catheter, 3.0 mm o.d., was introduced into the right femoral artery and advanced into the ascending aorta. A left thoracotomy was performed through the fourth intercostal space and the heart suspended in a pericardial cradle. A heparin-filled catheter (3.0 mm o.d.) was introduced into the left ventricle through the apical dimple and secured with a purse string suture. A similar catheter was inserted into the left atrium through the atrial appendage. A homemade intra-cardiac vein catheter (0.3 mm o.d) was inserted directly into the great cardiac vein for coronary vein blood sampling. A 1.5–2.0 cm segment of the proximal left anterior descending coronary artery (LAD) was dissected free and a hydraulic occluder constructed of polyvinyl chloride tubing (2.7 mm o.d.) was placed around the artery. A silicone elastomer catheter (0.75 mm i.d.) was placed into the LAD distal to occluder by the method of Gwirtz [8]. The region of the left ventricle that became cyanotic upon inflation of the occluder was determined by visual inspection and a 28 mm diameter MRS surface coil was sutured onto the pericardium overlying the ischemic area. The pericardial cradle was then released and the heart allowed to assume its normal position. The surface coil leads were connected to a balanced tuned circuit and the animals were placed in the magnet.

### MRS spectroscopy-general methods

Measurements were performed in a 40 cm bore 4.7 Tesla magnet interfaced with a SISCO (Spectroscopy Imaging Systems Corporation, Fremont, CA) console. The left ventricular pressure signal was used to gate MRS data acquisition to the cardiac cycle, while respiratory gating was achieved by triggering the ventilator to the cardiac cycle between data acquisitions [18], [20]. ^31^P and ^1^H-MRS frequencies were 81 MHz and 200.1 MHz, respectively.

### MRS deoxy-myoglobin methods

The method for ^1^H-MRS detection of the proximal histidyl N-δ proton resonance of Mb-δ has been described in detail [3]. Briefly, a single-pulse collection sequence with a Gaussian pulse (1 ms) was used to selectively excite the N-δ proton signal of the proximal histidyl of Mb-δ. This frequency selective pulse provided sufficient water suppression due to the large chemical shift difference between the water resonance and Mb-δ (>14 kHz). A short repetition time (TR = 35 ms) was used due to the short T_1_ value of Mb-δ. Each spectrum is acquired within 6 min (10,000 FID). Although the short T_1_ of Mb-δ and the fast acquisition prevent gating of data acquisition to the cardiac cycle, signal loss as a result of heart motion was negligible because of the inherently broad line width of the Mb-δ peak. Although the Mb-δ resonance is temperature sensitive, the chemical shift of this resonance which appeared at 71–72 ppm (relative to H_2_O), remained virtually constant during the study protocol. No other resonances were detected within a 10 ppm region. In phantom studies we have established that the detection sensitivity for Mb-δ is essentially flat across the wall of the left ventricle. Therefore, the Mb-δ resonance reflects average whole wall Mb-δ without the need for correction for differences in sensitivity in the deeper myocardial layers as is the case for HEP measurements (see below).

### Spatially localized ^31^P MRS technique

^31^P MR spectra were acquired in late diastole with a pulse repetition time of 6–7 seconds. This repetition time allowed full relaxation for ATP and Pi resonances, and approximately 90% relaxation for the PCr resonance. PCr resonance intensities were corrected for this minor saturation. RF transmission and signal detection were performed with a 28 mm diameter surface coil. A capillary containing 15 µl of 3M phosphonoacetic acid was placed at the coil center to serve as a reference. The proton signal of the water resonance was used to homogenize the magnetic field and to adjust the position of the animal in the magnet so that the coil was at or near the magnet and gradient isocenter. This was accomplished using a spin-echo experiment with a readout profile. The information gathered in this step was also utilized to determine the spatial coordinates for spectroscopic localization. Chemical shifts were measured relative to PCr which was assigned a chemical shift of −2.55 ppm relative to 85% creatine phosphate at 0 ppm. Spatial localization across the left ventricular wall was performed with the RAPP-ISIS/FSW method. The technical details of this method including voxel profiles, voxel volume, and the accuracy of spatial localization obtained in phantom studies and in vivo have been published elsewhere [9], [20], [21]. Briefly, signal origin was restricted using B_0_ gradients and adiabatic inversion pulses to an 18 mm×18 mm column coaxial with the surface coil and perpendicular to the left ventricular wall. Within this volume, the signal was further localized using the B_1_ gradient to 5 voxels spanning the left ventricular wall from epicardium to endocardium. Each set of spatially localized transmural spectra consisted of a total of 96 scans accumulated in a 10 minute block. Resonance intensities were quantified using integration routines provided by SISCO software. The values for PCr and ATP in each voxel were normalized to those present in the basal state and the PCr/ATP ratio was determined for each voxel. Pi resonances were also measured and ratio of Pi/PCr was calculated.

### Myocardial blood flow measurements

Myocardial blood flow was measured using radionuclide labeled microspheres, 15 µm in diameter labeled with 4 different radioisotopes (^51^Cr, ^85^Sr, ^95^Nb and ^46^Sc). Microsphere suspension containing 2×10^6^ microspheres was injected through the left atrial catheter while a reference sample of arterial blood was withdrawn from the aortic catheter at a rate 15 ml/min beginning 5 seconds before the microsphere injection and continuing for 120 seconds. Radioactivity in the myocardial and blood reference specimens was determined using a gamma spectrometer (Packard Instrument Company, Downers Grove, IL) at window settings chosen for the combination of radioisotopes used during the study. Activity corrected for overlap between isotopes and for background was used to compute blood flow as ml/g of myocardium/minute.

### Myocyte oxygenation

Myocyte oxygenation was estimated from the myoglobin oxygen saturation-PO_2_ relationship described in equation 1:
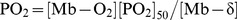
(1)


In equation 1, Mb-O_2_ and Mb-δ are fractional contents of oxy-myoglobin and deoxy-myoglobin, respectively, PO_2_ is the intramyocyte partial pressure of O_2_, and [PO_2_]_50_ is the partial pressure of O_2_ at which myoglobin is half saturated with O_2_ (at 37°C this is 2.38 mmHg) [22]. Therefore, if the integral of the Mb-δ resonance intensity measured during complete coronary occlusion represents total myoglobin content (in the Mb-δ form), then the resonance integrals determined during other experimental conditions can be normalized relative to this value to result in Mb-δ expressed as a fractional value of total myoglobin content. The fractional content of Mb-O_2_ during each experimental period can be calculated from the relationship Mb-O_2_  = 1 - Mb-δ. The fractional contents of Mb-O_2_ and Mb-δ and the temperature corrected [PO_2_]_50_ value can then be employed to calculate intracellular PO_2_ using equation 1. Because of continuing collateral blood flow we assumed that the Mb-δ resonance measured during total coronary occlusion represented 95% of the total myoglobin, and normalized the other Mb-δ resonances in relation to that value. We assumed that the fractional Mb-O_2_ content in the basal state (i.e., a time when no Mb-δ resonance was detected) was ∼90%. This also follows from calculations using equation 1. At 37°C, calculated intramyocyte PO_2_ would be 21 and 46 mm Hg for Mb-O_2_ saturations of 90% and 95%, respectively. Hence, we assumed that a fractional Mb-O_2_ content of ∼90% was present because this value is most compatible with the presence of an oxygen gradient between the capillary and intracellular myoglobin and reported values for coronary venous PO_2_ [10]. Because the present technique requires ∼10% fractional content of Mb-δ for detection [3] there is no experimental method to further test the latter assumption.

### Study protocol

Aortic and left ventricular pressures were measured with fluid filled pressure transducers positioned at mid-chest level and recorded on an 8-channel direct writing recorder (Coulbourne Instrument Company, Lehigh Valley, PA). Left ventricular pressure was recorded at normal and high gain for measurement of end-diastolic pressure. Hemodynamic measurements and ^31^P and ^1^H MRS spectra were first obtained under basal conditions.

**In protocol one**, of 6 dogs, baseline measurements were acquired at a FIO2 of 40% which leads to an arterial oxygen saturation of approximately 100%. This was followed by a two minute LAD occlusion. In the meantime the deoxymyoglobin MRS data were obtained for pO_2_ calculation. After the total occlusion Mb-δ data were acquired, FIO_2_ was progressively decreased to 21, 15, 10 and 5% respectively and interleaved P-31 and H-1 spectra obtained during each experimental condition every 4 minutes.

To examine if more severe hypoxia would produce an association between myoglobin desaturation and reduction of myocardial energetic state and alterations in OXPHOS, **in protocol 2**, 10 dogs (after baseline data were obtained) were exposed to 3% FIO_2_ and LAD occlusion, respectively. The P-31 and H1 MRS data were obtained similarly.

To examine whether the venous O_2_ would continue to decrease during prolonged hypoxia and whether this would be accompanied by a decrease in myocardial oxygenation level and PCr/ATP, **in protocol 3**, 9 dogs (after baseline data were obtained) were exposed to prolonged 18 minutes 5% FIO_2_, with P-31 and H1- MRS, myocardial blood flow and venous O_2_ measured every 4 minutes. Finally, the LAD was completely occluded (total occlusion) and all measurements were repeated.

### Data analysis

Hemodynamic data were measured from the chart recordings. ^31^P spectra were analyzed as described above. Transmural blood flow distribution was determined from the microsphere measurements. Data were analyzed with one-way analysis of variance for repeated measures. A value of p<0.05 was considered significant. When a significant result was found, individual comparisons were made using the method of Sheffé.

## Results

The hemodynamic, myocardial blood flow, arterial and coronary vein blood gas and myocardial oxygen consumption, myocardial HEP and oxygenation data are summarized in Tables 1, 2, 3, and 4, respectively.

**Table 1:** Hemodynamic Data.

	Heart Rate, beats/min	Mean Aortic Pressure, mmHg	LV Systolic Pressure, mmHg	LV End-Diastolic Pressure, mmHg	Rate-Pressure Product 1000x (mmHg.beats/min)
**Protocol 1**					
Baseline	130±15	86±7	108±10	6±1	14.4±2.8
21%	135±17	90±8	112±13	5±1	15.7±3.6
15%	136±20	87±11	111±14	6±2	15.7±4.0
10%	137±20	96±17	119±21	5±2	17.4±5.3
5%	135±16	116±17*	152±22*	6±2	21.5±5.4*
Occlusion	127±6	86±3	104±5*	6±1	14.0±1.0
**Protocol 2**					
Baseline	128±6	93±4	120±6	3±1	15.4±1.1
3%	124±9	128±9*	168±11*	7±1*	21.3±2.6*
Occlusion	130±8	90±4	110±5*	4±1	14.3±1.0
**Protocol 3**					
Baseline	132±19	103±6	124±5	4±1	16.2±2.2
FIO 5% 2mins	129±6	105±7	131±12	6±1	17.0±2.4
FIO 5% 4mins	127±7	113±11	140±14	5±1	18.0±2.9
FIO 5% 6mins	125±8	120±12	146±17	7±1	18.4±3.0
FIO 5% 8mins	122±6	123±10*	153±12*	6±1	18.8±2.5
FIO 5% 10mins	125±8	124±12*	163±18*	6±1	20.7±3.6*
FIO 5% 12mins	125±8	131±8*	175±11*	7±1	21.9±2.2*
Occlusion	128±4	88±4	105±4*	6±1	14.1±1.0

Values are means ± SD. * P<0.05 vs Baseline.

**Table 2:** Myocardial blood flow, oxygen consumption, and arterial - venous blood gas data.

	Epi	Mid	Endo	End/Epi	Mean Myocardial Blood Flow, ml.min-1.g-1	AV oxygen Difference, ml 02/100ml	MVO2, ml.min-1.100g-1	PH-AO	PH-CS	PaO2 (mmHg)	PcsO2 (mmHg)
**Protocol 1**											
Baseline	0.46±0.07	0.53±0.13	0.60±0.16	1.27±0.14	0.53±0.12	11.42±0.71	6.37±1.85	7.42±0.05	7.39±0.03	161±31	26±2
21%	0.62±0.12	0.63±0.18	0.68±0.23	1.05±0.19	0.64±0.17	8.01±1.79	5.94±2.62	7.43±0.05	7.40±0.04	55±9*	22±2*
15%	0.82±0.08*	0.91±0.10*	0.95±0.11*	1.14±0.12	0.89±0.10*	10.45±0.55	9.40±1.13*	7.51±0.03	7.46±0.03	53±1*	19±1*
10%	1.21±0.11*	1.44±0.14*	1.48±0.09*	1.20±0.13	1.38±0.18*	8.20±0.88	10.11±1.04*	7.47±0.02	7.44±0.02	38±4*	16±2*
5%	1.68±0.45*	1.87±0.56*	1.71±0.44*	1.01±0.09	1.75±0.48*	6.70±1.36*	10.06±2.03*	7.47±0.03	7.46±0.02	33±5*	14±3*
Occlusion	0.12±0.01	0.05±0.01	0.04±0.01	0.36±0.07	0.07±0.01	11.9±0.83	0.86±0.17	7.40±0.04	7.37±0.04	101±15	22±2
**Protocol 2**											
Baseline	0.45± 0.06	0.47±0.06	0.52±0.07	1.16±0.04	0.48±0.06	11.59±0.42	5.49±0.87	7.43±0.03	7.37±0.03	114±11	24±1
FIO 3%	1.09±0.16*	1.21±0.18*	1.22±0.19*	1.12±0.04	1.17±0.18*	4.18±0.43*	5.08±1.24	7.52±0.02	7.49±0.02	21±1*	12±1*
Occlusion	0.10±0.01	0.04±0.01	0.03±0.01	0.35±0.05	0.06±0.01	11.4±0.70	0.74±0.10	7.40±0.02	7.37±0.05	102±14	21±2
**Protocol 3**											
Baseline	0.57±0.06	0.56±0.05	0.58±0.04	1.05±0.04	0.57±0.05	10.28±0.72	5.92±0.69	7.36±0.03	7.33±0.02	156±19	30±2
FIO 5% (3mins)	1.35±0.11*	1.44±0.18*	1.43±0.12*	1.04±0.10	1.41±0.12*	7.81±0.73*	10.79±1.17*	7.48±0.04	7.47±0.03	37±4*	15±4*
FIO 5% (8mins)	2.92±0.26*	3.30±0.38*	3.00±0.29*	1.02±0.04	3.07±0.38*	3.43±0.33*	8.49±0.84*	7.46±0.03	7.44±0.04	21±2*	11±2*
Occlusion	0.10±0.02	0.05±0.01	0.03±0.01	0.35±0.06	0.06±0.01	11.6±0.83	0.80±0.14	7.40±0.04	7.37±0.05	103±12	21±3

Values are Mean±SD, * p<0.05 vs. Baseline.

**Table 3:** Myocardial PCr/ATP and Pi/PCr Data.

	Pi/PCr	CP/ATP	CP (Normalized)	ATP (Normalized)
**Protocol 1**				
BL	ND	2.34±0.10	1.00	1.00
FIO 21%	ND	2.37±0.31	0.93±0.04	0.94±0.04
FIO 15%	0.03±0.03	2.33±0.35	0.95±0.01	0.96±0.07
FIO 10%	0.04±0.03	2.25±0.23	0.92±0.03	0.97±0.03
FIO 5%	0.06±0.03	2.09±0.49	0.76±0.14	0.87±0.08
Total Occlusion	0.80±0.13	1.40±0.15†	0.45±0.04	0.77±0.03
**Protocol 2**				
Baseline	ND	2.34±0.10	1.00	1.00
FIO 3%	0.22±0.01	1.96±0.12*	0.73±0.03	0.84±0.02
Total occluded	0.78±0.10	1.35±0.10†	0.40±0.04	0.75±0.02
**Protocol 3**				
Baseline	ND	2.28±0.06	1.00	1.00
FIO 5% 4mins after	0.04±0.02	2.20±0.06	0.92±0.01	0.97±0.01
FIO 5% 8mins after	0.21±0.01	1.91±0.11*	0.77±0.05	0.91±0.02
FIO 5% 10mins after	0.33±0.04	1.83±0.13*	0.69±0.07	0.86±0.04
FIO 5% 14mins after	0.54±0.05	1.71±0.19*	0.62±0.08	0.82±0.05
Total occlusion	0.76±0.12	1.38±0.12†	0.42±0.04	0.76±0.02

Values are means ± SE. * P<0.05 vs. Baseline. † P<0.01 vs. Baseline.

**Table 4:** Deoxymyoglobin Data.

	Deoxymyoglobin
**Protocol 1**	
BL	0
FIO 21%	0
FIO 15%	0
FIO 10%	0
FIO 5%	0
Total Occlusion	1
**Protocol 2**	
Baseline	0
FIO 3% (2 min)	0.21±0.04*
FIO 3% (3.5 min)	0.26±0.03*
FIO 3% (5 min)	0.33±0.06*
FIO 3% (6.5 min)	0.45±0.07*
FIO 3% (8 min)	0.57±0.04*
Total occluded	1
**Protocol 3**	
Baseline	0
FIO 5% (2 min)	0
FIO 5% (3.5 min)	0
FIO 5% (5 min)	0
FIO 5% (6.5 min)	0
FIO 5% (8 min)	0.26±0.06*
FIO 5% (10 min)	0.29±0.06*
FIO 5% (14 min)	0.39±0.07*
FIO 5% (16 min)	0.50±0.05*
Total occluded	1

Values are means ± SE. * P<0.05 vs. Baseline.

### Protocol 1

**Figure 1:**
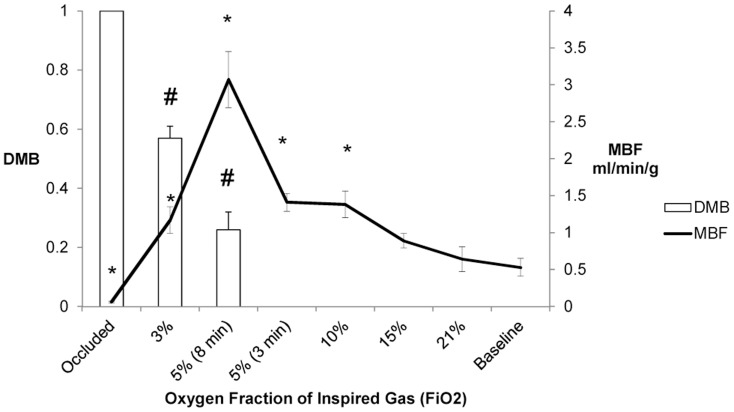
Relationship of Myocardial Blood Flow and Tissue Oxygenation with Oxygen Fraction of Inspired Gas. DMB  =  level of deoxymyoglobin normalized to total LAD occlusion; MBF  =  myocardial blood flow rate (ml per minute per gram myocardium measured by microspheres at each experimental conditions). *, p<0.01 VS Baseline; #, p<0.01 VS. LAD occlusion.

Hemodynamic measurements during each experimental condition are shown in Table 1. Heart rate and LV systolic pressure remained unchanged during progressively more severe hypoxia (21%, 15%, 10%) until 5% FIO_2_ when mean aortic pressure and LV systolic pressure increased with subsequent increase in RPP [Table 1]. Even though the arterial venous oxygen difference decreased with progressive reduction in FIO_2_, MVO_2_ actually increased [Table 2] and this was related to an increase in MBF with decrease in FIO_2_ (1.75±0.48 ml/min/g at 5% vs 0.53±0.12 ml/min/g at baseline, p<0.05). However, myocardial HEP remained relatively stable [Table 3]. Mb-δ resonance was undetectable at baseline, and remained so until the LAD occlusion experimental condition, in which a prominent Mb-δ resonance appeared as expected [Table 4 and figure 1].

### Protocol 2

To examine whether more severe hypoxia would produce an association between myoglobin de-saturation and reduction of myocardial HEP, 10 dogs were exposed to 3% FIO_2_. The mean aortic pressure and LV systolic pressure increased substantially resulting in a significant increase in rate pressure product [Table 1]. MVO_2_ increased [Table 2] again as a result of the increase in MBF, but this was not sufficient to maintain the baseline energetic state [Table 3]. Coronary venous O_2_ decreased to 12±0.9, which is significantly lower than the myocardial ischemia induced increase of oxygen extraction. The deoxymyoglobin reached approximately 50% of maximum deoxymyoglobin level [Table 4].

### Protocol 3

Protocol 3 was performed to examine whether prolonged 5% FIO_2_ would result in continuous reduction of coronary venous pO_2_ and therefore detectable Mb-δ. Prolonged 5% FIO_2_ caused a significant increase in LV systolic pressure and subsequent RPP. MVO_2_ increased as a result of increase in MBF but there was a significant decrease in HEP which was associated with an increase in deoxymyoglobin after 8 minutes exposure. It was evident that when the 5% FIO_2_ was maintained the venous pO_2_ continued to decrease. Once it was below 14 mm Hg, the Mb-δ became detectable.

### Myocyte oxygenation and HEP levels

**Figure 2:**
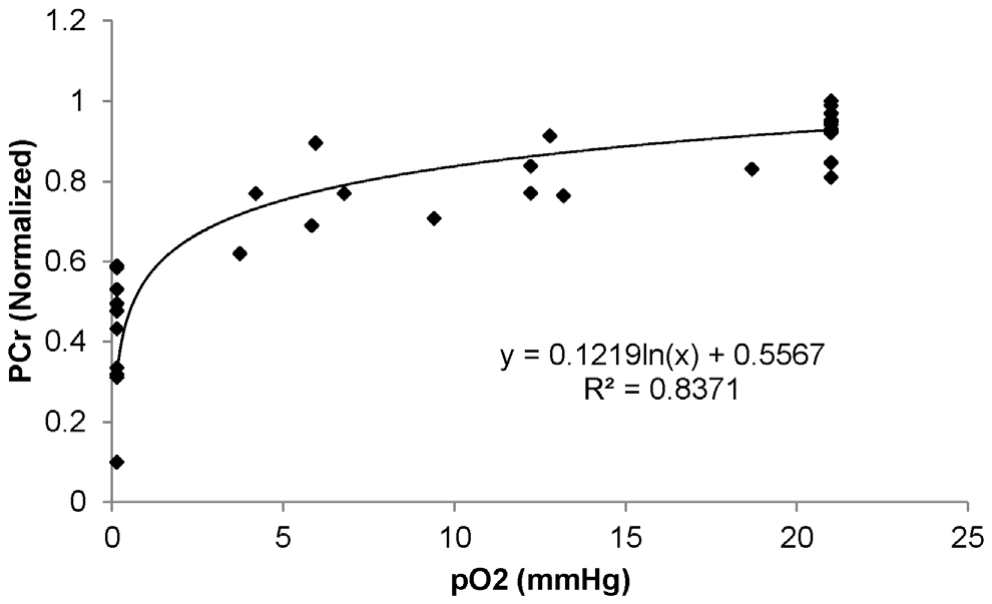
Relationship of PCr with pO_2_.

**Figure 3:**
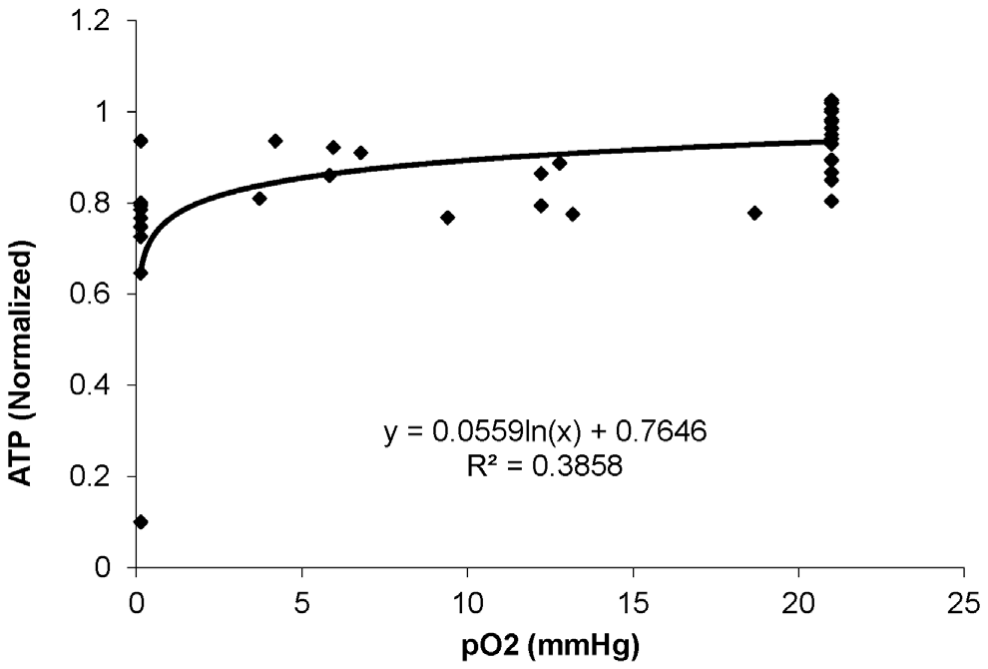
Relationship of ATP with pO_2_.

Decreasing FIO_2_ beyond 5% or prolonged exposure to low FIO_2_ was associated with a decrease in PCr/ATP and increase in Pi/PCr which was associated with a decrease in intracellular PO_2_. The relationships of PCr and ATP with intracellular PO_2_ are shown in Fig 2 and 3 respectively. These graphs were obtained assuming that the Mb-δ resonance measuring total coronary occlusion represented 95% of the total myoglobin and that the fractional myoglobin O_2_ content in the basal state was 90%. Values for PCr decreased when intracellular PO_2_ approached 10 mmHg. PO_2_ values that are associated with significant reductions of HEP have been termed “critical” with the implication that they reflect the presence of O_2_ limitation of oxidative phosphorylation. Based on Fig 3, the PO_2_ value for 20% and 50% reduction of PCr were approximately 7.4 and 0.6 mmHg respectively. Similarly, the (PO_2_)_80_ for ATP was approximately 1.9 mmHg. A (PO_2_)_50_ value for ATP could not be determined because this degree of ATP reduction was not achieved. The lower critical PO_2_ for ATP than for PCr is not surprising because the fall of ATP is buffered by PCr through the creatine kinase reaction.

## Discussion

The present study examined whether mitochondria lack oxygen under the extreme low FIO2 conditions, and if so, how long it takes the compensation systems to fail. We examined whether the ^1^H- Mb-d and ^31^P- MR spectroscopic measurements can detect the signals of reduction of oxygen delivery to the mitochondria. Prior studies did not measure Mb-d, therefore, no evidence of decrease of oxygen delivery to the mitochondria under these conditions. The findings from the present study demonstrate that the in vivo compensate mechanisms can maintain the normal mtOXPHOS even during extreme low FIO2. The present study has further elucidated the response of the myocardial oxidative phorsphorylation (OXPHOS) regulation to hypoxia. Firstly, it has reiterated the hemodynamic response of the in vivo heart to graded hypoxia as a result of a decrease in the fractional inspired oxygen concentration. Secondly, it has depicted the increase in transmural blood flow during hypoxia, which helps in maintaining a normal mitochondrial OXPHOS. Thirdly, it has defined the relationships between myocyte PO_2_ and HEP levels in vivo myocardium under conditions where myocardial flow is not limited.

During myocardial hypoxia, the lactate production and glycolysis pathway of ATP production are increased [16]. The myocardial bioenergetic changes during hypoxia are characterized by the increase glucose uptake and lactate production. However, the lactate oxidation and free fatty acid oxidation associated ATP production in mitochondria remain significantly higher than the anaerobic glycolysis ATP production [16], [17].

Our study shows that the mean arterial pressure, heart rate, left ventricular systolic and end diastolic pressure and rate pressure product are maintained with graded reductions in FIO_2_ up to 10% [Table 1]. With further reductions in inspired oxygen concentration, mean arterial pressure, left ventricular systolic pressure and consequently RPP increases [Table 1]. These changes in hemodynamics are similar to a previous study in a sheep model in vivo, in which graded reductions in FIO_2_ caused no changes in systemic hemodynamics up to 6% [19]. Further decrease actually caused an increase in cardiac power which was thought to be related to increased circulating catecholamines as a result of hypoxia [19]. In contrast, with LAD occlusion, RPP actually decreases [Table 1].

The graded hypoxia caused a proportional increase in myocardial blood flow up to 5% FIO_2_ [Table 2]. This was associated with maintenance of the endocardial/epicardial blood flow as opposed to a decrease in this ratio as occurs in coronary stenosis [Table 2]. However, further decrease in FIO_2_ caused a decrease in myocardial blood flow but it was still higher than baseline. This increased myocardial blood flow caused myocardial oxygen consumption to be maintained throughout hypoxia, as opposed to LAD occlusion [Table 2].

Under normal baseline conditions, the myocardium has adequate supply of oxygen for oxidative phosphorylation and oxygen does not seem to play a regulatory role. Even at high cardiac work states, no deoxymyoglobin signal is detected despite a decrease in high energy phosphates [26]; this implies that even at these increased cardiac work states, oxygen availability is non-limiting. However, if oxygen availability is reduced under pathological conditions, a point is reached where it becomes a limiting factor for oxidative phosphorylation and leads to decreased high energy phosphates. Hypoxia is caused by decreased oxygen content in the blood either due to anemia (decreased hemoglobin concentration) or due to hypoxemia (decreased arterial pO_2_). The effect of hypoxia on myocardial HEP has been studied in both of the above mentioned conditions in isolated working rat hearts perfused with Krebs-Henseleit buffer at 25°C [12], [13]. Using progressive reductions of the perfusate flow rate, Kreutzer and Jue showed that perceptible decrease of PCr occurred only when coronary flow rates were decreased sufficiently to reduce intracellular PO_2_ below 1.5 mmHg, with (PO_2_)_80_ of 1.1 mmHg and (PO_2_)_50_ of 0.5 mmHg [12]. However, the same group used progressive reductions in perfusate PO_2_ while coronary flow rate was maintained constant, and demonstrated that PCr began to decrease at an intracellular PO_2_ of 2 mmHg, with a (PO_2_)_80_ of 1.8 mmHg [13] which was higher than the value obtained by reductions in flow rates. In the in vivo setting using graded coronary stenosis, our laboratory previously found that PCr began to decrease with PO2<5 mmHg with (PO_2_)_80_ of 4.3–4.5 and (PO_2_)_50_ of 1.0–1.2 mmHg [27]. It is possible that reduced metabolic rates of rat hearts perfused at 25°C compared with normothermic in vivo conditions could contribute to a lower critical PO_2_ for PCr in the perfused heart. As the critical PO_2_ in the perfused rat hearts is higher in the graded perfused PO_2_ setting as compared to graded coronary flow setting, we hypothesized that we may find a similar trend in the in vivo setting. In the present study, we examined the critical PO_2_ in the vivo setting with graded hypoxia and found that the (PO_2_)_80_ is 7.4 mmHg, which is significantly higher than the hypoperfusion setting.

The higher PO_2_ required for decrease in HEP in the present study suggests that there were other factors which are affecting the intracellular PO_2_ in the graded coronary artery stenoses setting. Indeed, the response of myocardial HEP levels to decreased coronary perfusion can be affected by changes in myocardial oxygen demand. Gregg initially described that myocardial oxygen consumption can be influenced by coronary flow and perfusion pressures. Several mechanisms have been proposed to modulate oxygen demand during hypoperfusion. The pressure in the coronary system may distend the ventricles, leading to increased contractility and oxygen consumption by an increase in sarcomere length or ventricular stiffness [1], [25]. Marban *et al* showed that in the range of coronary pressure of 60–160 mmHg, decreases in coronary perfusion pressure in isolated ferret hearts resulted in preload independent decrease of the calcium transient which they hypothesized represented a protective mechanism that minimizes energy demands during low flow ischemia [15]. This has subsequently been shown in intact animals and has been termed as the early phase of myocardial hibernation. In open chest studies of swine or dogs, 30–40% reductions in coronary flow initially caused a decrease of myocardial PCr, but this was followed by recovery to near normal levels within 60 minutes despite persistent hypoperfusion [5], [18]. Thus, stenosis induced hypoperfusion leads to decreased myocardial oxygen consumption through signals that decrease contractile activity or even by signals that directly inhibit mitochondrial respiration. The decreased oxygen consumption allows the myocardium to maintain HEP levels at lower intracellular PO_2_ levels. Thus, the true critical PO_2_ level that leads to a reduction of HEP can be better estimated with graded hypoxia through decrease in FIO_2_ such as in the present experimental setting. This reveals a (PO_2_)_80_ of 7.4 mmHg for PCr and a (PO_2_)_80_ of 1.9 mmHg for ATP.

Another important question to address is why any oxygen limitation occurs at an intramyocyte PO_2_ that far exceeds the apparent Michaelis-Menten constant of cytochrome oxidase with respect to O_2_. It was shown that the critical PO_2_ at which oxidative phosphorylation began to decrease in isolated mitochondria was approximately 0.01 mmHg, which is more than 100 fold lower than the PO_2_ at which HEP levels started to decrease in the present study. This could be attributed to inhomogeneities of O_2_ availability at the cellular level, if the PO_2_ gradient between the cytosol and the inner mitochondrial membrane was significant. However, mathematical models of O_2_ diffusion suggest very low perimitochondrial O_2_ gradient [4]. Furthermore, high resolution spectrophotometric measurements performed on single cardiomyocytes have demonstrated a steep spatial PO_2_ gradient near the sarcolemma, while PO_2_ values toward the center of the cell are nearly flat [24]. However, Ito *et al* postulated that since the mitochondrial membrane is impermeable to myoglobin, a significant PO_2_ gradient can occur because oxygen must diffuse through a myoglobin free volume before it can react with cytochrome oxidase [11]. Recently, myoglobin facilitated oxygen diffusion has also been shown to play a role in oxygen flux. Lin *et al* have recently shown that in the myocardium, Mb-facilitated O_2_ diffusion contributes increasingly more than free O_2_ diffusion when the PO_2_ falls below 1.77 mmHg [14]. Thus, under normal condition where myocardial PO_2_ is around 10 mmHg, myoglobin facilitated oxygen diffusion does not play a major role but under hypoxic conditions such as in the present study it plays a significant role in oxygen diffusion.

In conclusion, during graded hypoxia caused by decreased FIO_2_, significant loss of PCr occurred at an intracellular PO_2_ of 7.4 mmHg. Thus, in normal in vivo heart, oxygen availability plays an important role in regulation of oxidative phosphorylation only when mean intracellular PO_2_ falls below 7.4 mmHg.
